# Implementing internet- and tele-based interventions to prevent mental health disorders in farmers, foresters and gardeners (ImplementIT): study protocol for the multi-level evaluation of a nationwide project

**DOI:** 10.1186/s12888-020-02800-z

**Published:** 2020-08-27

**Authors:** Johanna Freund, Ingrid Titzler, Janika Thielecke, Lina Braun, Harald Baumeister, Matthias Berking, David Daniel Ebert

**Affiliations:** 1 Department of Clinical Psychology and Psychotherapy, Institute of Psychology, Friedrich-Alexander University of Erlangen-Nürnberg, Erlangen, Germany; 2 GET.ON Institute, Berlin, Germany; 3 Department of Clinical Psychology and Psychotherapy, Institute of Psychology and Education, Ulm University, Ulm, Germany; 4 Department of Clinical, Neuro- & Developmental Psychology, VU University Amsterdam, Amsterdam, Netherlands

**Keywords:** Implementation, Internet- and tele-based interventions, RE-AIM framework, Prevention, Mental health, Farmers

## Abstract

**Background:**

Farmers are a vulnerable population for developing depression or other mental health disorders due to a variety of risk factors in their work context. Beyond face-to-face resources, preventive internet- and tele-based interventions could extend available treatment options to overcome barriers to care. The German Social Insurance Company for Agriculture, Forestry and Horticulture (SVLFG) implements several guided internet- and mobile-based interventions and personalised tele-based coaching for this specific target group provided by external companies within a nation-wide prevention project for their insured members. The current study aims to evaluate the implementation process and to identify determinants of successful implementation on various individual and organisational levels.

**Methods:**

The current study includes two groups of participants: 1) insured persons with an observable need for prevention services, and 2) staff-participants who are involved in the implementation process. The Reach, Effectiveness, Adoption, Implementation and Maintenance (RE-AIM) framework and the Consolidated Framework for Implementation Research (CFIR) will be used to track and evaluate the implementation process. A mixed-method approach will provide insights on individual and organizational level (e.g. degree of normalization, readiness for change) and helps to identify determinants of successful implementation. In-depth insights on experiences of the participants (e.g. acceptance, satisfaction, barriers and facilitating factors of intervention use) will be yielded through qualitative interviews. Focus groups with field workers provide insights into barriers and facilitators perceived during their consultations. Furthermore, intervention as well as implementation costs will be evaluated. According to the stepwise, national rollout, data collection will occur at baseline and continuously across 24 months.

**Discussion:**

The results will show to what extent the implementation of the internet- and tele-based services as a preventive offer will be accepted by the participants and involved employees and which critical implementation aspects will occur within the process. If the implementation of the internet- and tele-based services succeeds, these services may be feasible in the long-term.

**Trial registration:**

German Clinical Trial Registration: DRKS00017078. Registered on 18.04.2019.

## Background

Depressive disorder is a common condition with a 12-month prevalence rate of 8.56% in European countries [1]. Previous research has shown that farmers [2–5] and employees in related occupations (e.g. forestry and fishery workers) [6] are at an increased risk for developing mental health problems, especially depression, compared to non-agricultural occupations. Risk factors for mental disorders associated with agricultural jobs include: work-related stress [7, 8], longer work hours [3], poor harvest and weather [2], financial problems [9], health problems [8, 10] and exposition to pesticides [8].

At the same time, farmers mainly live in rural areas with limited mental health care. In Germany, the availability of on-site psychotherapy is additionally restricted by long waiting times in rural areas [11, 12]. Furthermore, stigma surrounding mental disorders and resistance to mental health care are reported in rural areas [5]. Especially male farmers are often constrained to friends and family for mental health support, while professional face-to-face therapeutic treatments are rarely obtained [5]. Thus, alternative ways to deliver treatment might be beneficial for farmers [2].

Existing evidence-based treatments for mental disorders have shown to only reduce the burden of mental health disorders by a third [13]; however, symptoms of mental disorders can be effectively reduced with preventive interventions [14, 15]. As a recent meta-analysis showed, psychological interventions can prevent or delay the onset of Major Depressive Disorder (MDD) by focusing on individuals with increased risk (selective prevention) or with subclinical depressive symptoms (indicated prevention) [16].

### Internet- and tele-based interventions

Easily accessible preventive interventions, as implemented in this study, could increase farmers’ ability to overcome reported challenges. Internet- and mobile-based psychological interventions (IMIs) have been shown to be effective in working participants [17] as well as for preventing MDD onset and treating subthreshold depression in the general population [16, 18–21]. In a similar context, researchers have begun to evaluate online interventions in Australian farmers to reduce stigma surrounding suicide and to combat rural isolation for farmers [22].

Although access to the internet has increased significantly in Germany in recent years, rural areas still face problems using Internet services [23]. Therefore, tele-based coaching is an important addition to existing preventive programmes against depression, especially in rural communities. Evidence also suggests that tele-based coaching, in comparison to treatment-as-usual, is effective in reducing symptoms of depression [24].

Despite its effectiveness, the uptake of health care interventions in routine practice remains low and costly [25, 26]. Only 14% of evidence-based health care interventions are used in routine care [27]. While many studies focus on the implementation of interventions for the treatment of mental disorders, there is little research on the implementation of preventive interventions. The few studies relate to the prevention of eating disorders and depression in adolescents and young adults [28, 29].

### Implementation in rural areas

Technology-based solutions appear to be an increasingly viable option as they have the potential to overcome disparities in access for rural mental health care services [30, 31]. In addition, computerised Cognitive Behavioural Therapy interventions for anxiety and/or depression seem to be acceptable among people living in rural and remote areas [32]. Moreover, barriers and facilitating factors for the implementation of health care interventions in rural areas have been studied: Current evidence suggests that close collaborations with professional institutions, training of employees and perceived credibility of the treatment may facilitate successful interventions [33]. These facilitating factors may be achieved by supporting government agencies, qualified program managers or empirical evidence of efficacy [33]. Furthermore, increased public awareness for mental disorders [34] as well as for the benefits of telehealth [35] might be beneficial.

In addition to the participants, perspectives of all people involved in the implementation and delivery of this treatment should be considered. For example, health care staff in rural areas may prioritise staff attitudes and beliefs towards treatment [36, 37] as well as the leadership and organisational structures of the clinic and the community [37]. Likewise, organisational readiness within rural communities is imperative for the successful implementation of telehealth services [38].

Implementing mental health services in rural areas faces specific difficulties since existing studies disregard the diversity of rural settings, are rarely based on well-developed theories and show methodological weaknesses [39]. Studies exist regarding particular aspects of implementation in rural areas (e.g. acceptance of interventions). However, there is a lack of comprehensive, multi-level implementation research in routine care, especially with regards to the implementation of technology-based interventions for mental disorders in rural areas.

### A nationwide pilot project

This study is part of a national depression prevention program for farmers, gardeners and foresters (“With us in balance”) carried out by the German Social Insurance company for Agriculture, Forestry and Horticulture (SVLFG, www.svlfg.de). The study aims to evaluate the implementation of tailored IMIs and a personalised tele-based coaching as an extension to existing face-to-face group interventions. In addition to the implementation study, it is evaluated in parallel running, randomised controlled trials whether the internet- and tele-based interventions are (cost-)effective in reducing depressive symptoms and other mental health concerns as well as in preventing the onset of clinical depression in this specific target group of farmers, gardeners and foresters [40, 41].^1^

### Evaluation frameworks

Implementation success is considered as a multi-dimensional construct. In this study, the evaluation is based on the Reach, Effectiveness, Adoption, Implementation and Maintenance (RE-AIM) framework [42] as it helps to structure this multifaceted evaluation on individual and organisational levels. The RE-AIM framework is one of the most used evaluation frameworks in the implementation setting and facilitates to assess the quality, speed and impact of implementation efforts. In addition, the use of the Consolidated Framework for Implementation Research (CFIR) enables a deeper understanding of the mentioned ‘implementation’ dimension, as CFIR offers a pragmatic structure for approaching compound and interacting states in the implementation of interventions [43].

Staff are part of a dynamic interaction within the organisation and are a key component to successful implementation. Previous literature showed the importance to examine different levels, when implementing in rural areas, e.g. staff attitudes and beliefs [36, 37], management and organisational structures, and organisational readiness [38]. The following constructs focusing on the involved employees aim to capture different aspects of the implementation process:

- Degree of Normalisation: Normalisation refers to activities that people pursue to integrate an innovation into their everyday routine. According to the Normalisation Process Theory, normalisation is defined as the extent to which a new intervention is considered as a normal part of daily work [44].
- Determinants in the implementation process: The Theoretical Domains Framework (TDF) provides an integrative framework model to identify drivers of behavioural change that could influence determinants in the implementation process [45].
- Organisational leadership: Minimal attention is given in research to the organisational context, although it may have a significant impact on the implementation of evidence-based practices [46]. Therefore, this study collects information regarding implementation leadership which may have an impact on the program’s ability to drive change and innovation [47].
- Organisational readiness for change: This construct refers to the extent to which employees of an organisation are willing to implement organisational change, with particular focus on psychological and behavioural aspects [48].

### Research questions

The aim of this study is the multi-level evaluation of the implementation success and stepwise nationwide rollout of the following new preventive services: a) GET.ON online health trainings with 7 guided IMIs, and b) personalised tele-based coaching provided by external companies in addition to existing on-site group interventions offered by the SVLFG.

Our study seeks to address the following research questions:

- How successful is the implementation of internet- and tele-based interventions in terms of reach, effectiveness, adaptation, implementation and maintenance on an individual (participants, staff) and organisational level?
- How effective is the implementation in terms of intervention uptake, normalisation into everyday life, and implementation costs?
- What are the experiences and impressions of the participants receiving internet and tele-based interventions? In particular, to what degree do participants report: a) acceptance and use of the technology-based treatment, b) satisfaction with the intervention considering their expectations, and c) perceived barriers and facilitators to use the intervention?
- Which barriers and facilitating factors can be identified for advising on prevention services at staff level?
- What determinants for implementation success in rural areas can be identified by focusing on employees involved in the referral process?
- Which similarities and differences can be identified between internet- and tele-based interventions with regards to implementation success and other determinants metrics of implementation effectiveness?

## Methods

### Design of the study

This study is a prospective, longitudinal implementation study and is described according to the Standards for Reporting Implementation Studies (StaRI) checklist [49], see Supplementary material. A mixed-methods approach using qualitative interviews, focus groups, quantitative surveys and reporting data is utilised for a comprehensive understanding of the implementation process. The evaluation is based on the RE-AIM framework [42] and the CFIR [43].

**Fig. 1:**
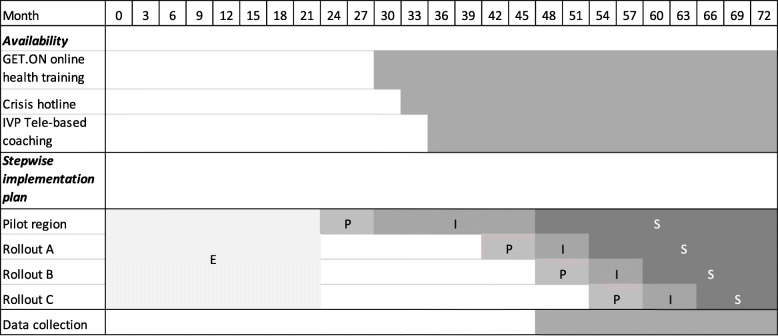
Project timeline depicting the Conceptual Model of Implementation Phases [50] including exploration (E), preparation (P), active implementation (I) and sustainment (S) adapted to the stepwise rollout plan

The implementation follows a stepwise, nationwide rollout plan which determines the availability of the internet- and tele-based interventions and the enrolment of the insured persons in each rollout region. Each region (2–7 federal states of Germany) undergoes these implementation phases at different times, starting with a pilot implementation phase in Bavaria and Schleswig-Holstein for 1.5 years and following with three rollouts (A, B and C) over 2 years. The initial pilot implementation phase was extended for various reasons (e.g. additional time needed to implement internal structures at SVLFG such as the referral process and documentation of consultations with insurers, additional time to conduct trainings, and unforeseen delayed availability of the tele-based coaching). Based on the Conceptual Model of Implementation Phases [50], the study can be divided into the following phases: exploration, preparation, implementation (6–12 months) and maintenance (12–24 months). The duration of the phases was defined in accordance to the RE-AIM framework that recommends 6 to 12 months for the implementation phase and at least 2 years for the maintenance phase [42]. In addition, the enrolment plan of the SVLFG stipulates that the rollout area is expanded every 6 months and focuses on implementation in this region within these 6 months Fig. 1.

### Enrolment

The insured individuals are advised on the preventive services by approximately 350 employed field workers and in-house staff of SVLFG during their consultations via phone or on-site. In addition, the prevention services are advertised widely through PR activities of the SVLFG (e.g. newspaper articles, radio reports, SVLFG member magazine, lectures and information events) in the federal states where the interventions have been implemented. Interested individuals receive detailed information regarding the offered prevention services and their eligibility at the SVLFG central call centre. Eligible insured persons can choose whether they prefer single (e.g. internet- or tele-based interventions) or group preventive services. The group interventions are delivered on-site and include various topics on mental health issues for this target group (e.g. dealing with stress, handing over the business to the next generation). The registration process is conducted by SVLFG.

Data collection starts with rollout A in April 2019 and is expected to be finished at the project end (April 2021).

### Interventions

The internet- and tele-based interventions are described according to the *Template for intervention description and replication* (TIDieR) [51], see Supplementary material. They are briefly outlined below.

#### Tailored IMIs

**Fig. 2:**
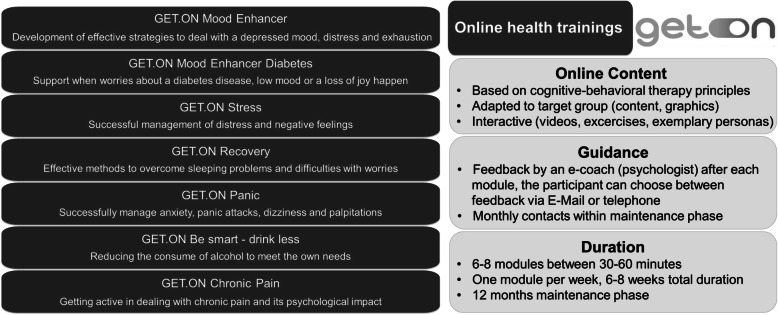
Online content, guidance and duration of the GET.ON online health trainings

Seven IMIs are provided by the GET.ON Institute (www.geton-institut.de). Their effectiveness in the prevention and treatment of mental disorders has been demonstrated in more than 30 randomised controlled trials [19, 20, 52–65]. The IMIs are individually tailored to the participants based on their symptoms, risk profile and needs, and follow the selective prevention approach (e.g. providing trainings for a group with increased risk for mental disorders). The different IMIs are addressing risk factors for the incidence of depression including subclinical depressive symptoms [66], insomnia [67], stress [68], anxiety [69], chronic pain [70], harmful alcohol use [71, 72] and subclinical depressive symptoms in the context of diabetes [73]. The IMIs are adapted to the target group of farmers, foresters and gardeners in terms of content (e.g. descriptions of mock individuals facing similar struggles) and graphics to meet the needs of the participants. Variation of content and duration of the seven IMIs is illustrated in Fig. 2.

To begin the intervention phase, the participant completes a computer-adaptive psycho-diagnostic assessment which is based on self-report questionnaires assessing mental health issues. The type of online training is chosen during the initial interview with the e-coach depending on the participant’s symptom profile, needs and preferences. The modules include psychoeducation, testimonials of mock individuals, in-depth exercises, a diary and homework to improve mental health and wellbeing. Depending on the training type, participants have access to additional modules. After each module, the participant receives individual feedback by email or by phone from the e-coach which helps to improve adherence of e-health interventions [74].

The e-coaches are psychologists with a university degree who have at least started a psychotherapeutic training. The training of the e-coaches consists of supervision by licensed psychotherapists in CBT training and information about IT-matters. A detailed description is published in the study protocols elsewhere [40, 75–80].

#### Personalised tele-based coaching

The personalised tele-based coaching is provided by the company IVPNetworks (www.ivpnetworks.de) and focuses on general mental health problems. The coaches are psychologists with trainings either in cognitive behavioural therapy, systemic therapy, psychodynamic psychology, hypnotherapy or other therapeutic trainings. Licensed psychotherapists are responsible for supervision. The coaches support participants in recognising and understanding conflict patterns in order to effectively cope with issues by activating their own resources. This is pursued by approaching the participant’s personal situation and stressors (e.g., financial burden, family problems, work-related stress).

**Fig. 3:**
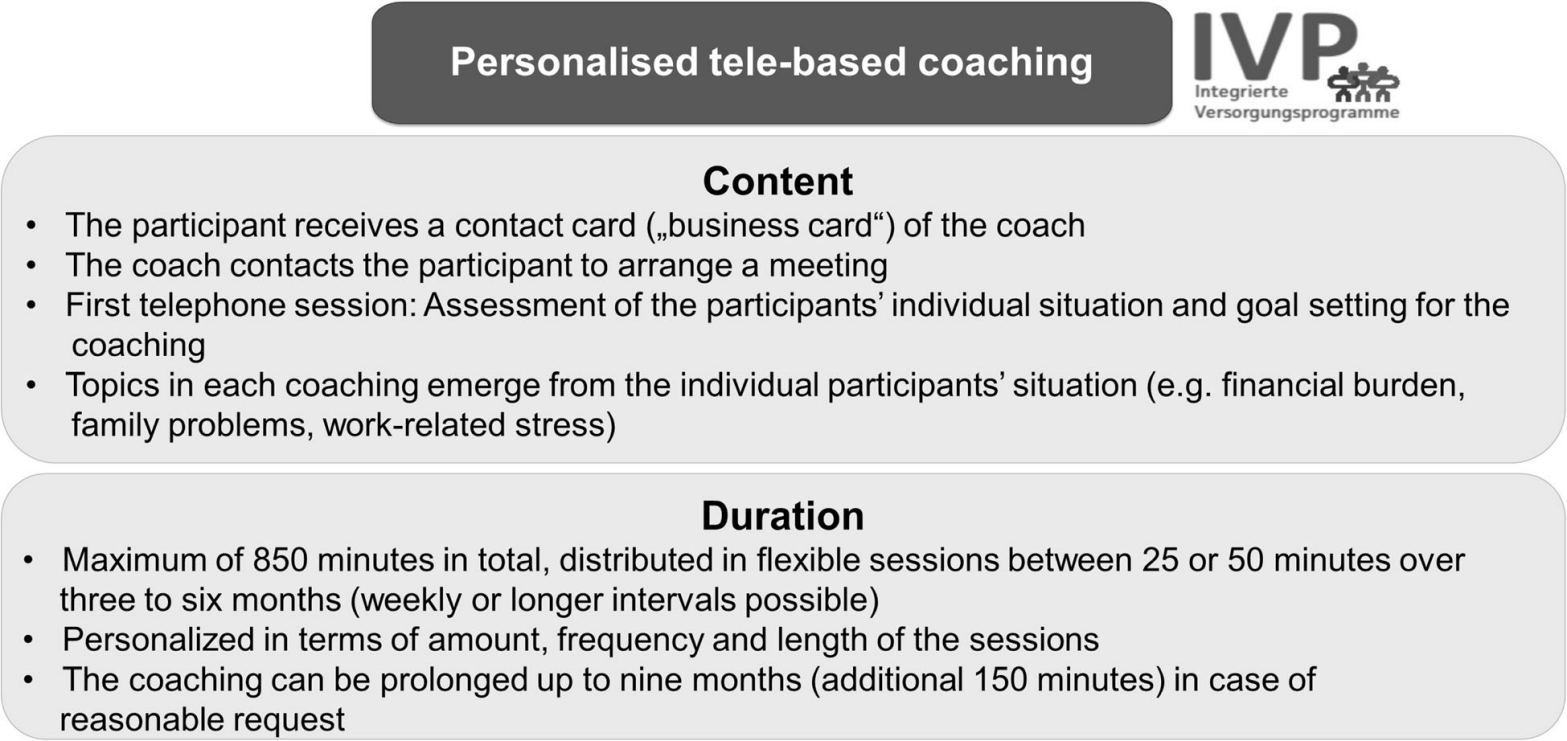
Content and duration of the personalised tele-based coaching at IVPNetworks

Participants are registered with their contact information in the IVPNetworks management and documentation platform (IVPnet 2.0). A case manager at IVPNetworks assigns the participant to a coach. The coaching is personalised in terms of amount, frequency and duration of the sessions, depending on the individual participant’s needs. Personalised coaching means that there are no standardised manuals or fixed procedures for the coaching. Different therapeutic methods are used depending on the therapeutic background of the coach (which will be monitored in the study). If indicated, participants are supported in finding on-site social and health care services to complement the tele-based coaching (e.g., socioeconomic consultants, agricultural family counselling). Alternatively, on-site coaching can be arranged if a participant no longer prefers tele-based coaching. Common content and duration of the personalised tele-based coaching at IVPNetworks are illustrated in Fig. 3.

In addition to the personalised tele-based coaching, IVPNetworks offers a crisis hotline that can be used 24/7 by those insured at SVLFG. The crisis hotline is managed by trained psychologists and is a resource for acute psychological emergencies and crisis situations. As part of the crisis hotline, up to five follow-up contacts are possible.

### Participants

The study design includes two groups of participants involved in the implementation process:

#### Group 1: insured persons at SVLFG

Inclusion criteria for insured persons at SVLFG includes: being an insured member of the health insurance scheme OR the pension fund of the SVLFG, being an agricultural contractor OR a collaborating spouse OR a family member or a pensioner, being over 18 years old, having access to the internet and/or a telephone, and providing informed consent.

Persons will be excluded if they currently suffer from a mental disorder and need extensive therapeutic service. Individuals may be excluded during the consultation in the call centre (if the interested insured person reports a mental disorder), during the assessment by the service provider or during their participation in case of a suspected mental disorder. If there is a suspicion, the external service provider advises the participant that a consultation with a general practitioner is recommended. The insured individual gets support in the search for further treatment options. In addition, people can be excluded for the tele-based intervention, if they have substantial hearing impairment or for the online interventions, if they have a visual impairment.

There are 465,000 eligible insured members of SVLFG nationwide, and the sample size is not capped. The number of participants enrolled in this study depends on the rollout stage of the implementation. Eligible insured persons can choose the specific intervention based on their preference and needs which reflects the conditions of routine care. A sample size of *N* = 100 per intervention is desirable to achieve reliable results. Based on previous experiences in the pilot phase of the implementation in two federal states, we expect a sample size of approximately 500 to 800 participants for the national implementation area.

#### Group 2: staff-participants at SVLFG, GET.ON and IVPNetworks

##### Field worker, in-house staff and call centre agents at the implementing company SVLFG

Approximately 350 field workers visit the insured members on-site. In-house staff counsel the insured persons by telephone. Both field workers and staff advise insured members on technical prevention, rehabilitation, etc. and additionally inform and advise members regarding the internet- and tele-based prevention services. They also forward interested persons to a nationwide call centre. Depending on the rollout phase (which includes a training on all health services offered by the SVLFG), the field workers begin advising members on the internet- and tele-based interventions at different times. Twenty-five call centre agents advise the insured individuals on various health offers including the newly implemented internet- and tele-based interventions. Additionally, call centre agents check the insurance eligibility criteria and are responsible for the enrolment in the prevention services.

Thus, implementation of internet- and tele-based interventions requires that these employees undergo changes to their work environment, especially at the behavioural level, by adapting their previous working routines to the new requirements.

##### Implementation team of SVLFG

Members of the implementation team are responsible for the planning and realisation of the implementation activities. Employees who work in two or more roles (e.g. as a field worker, in-house staff and/or call centre agent) are excluded from the staff surveys for field workers, in-house staff and call centre agents to avoid conflation of the data, as such persons cannot perform both roles simultaneously without bias.

##### Coaches at GET.ON and IVPNetworks

Coaches are involved in the delivery of the internet- or tele-based interventions and work as psychologists or therapists on a freelance basis or employment contract for GET.ON or IVPNetworks.

Approximately 150 staff-participants at GET.ON, IVPNetworks and SVLFG are expected to participate in the study. All employees of the described staff-groups who sign the informed consent are able to participate in the study. There are no specific eligibility criteria. If additional professional groups become involved in the future course of the implementation of these services, it will be decided whether these employees will be included in the surveys.

### Measurements

The used measures are summarised in Table 1, followed by a description of each RE-AIM dimension and selected outcomes. Measurements include routine data, staff reviews of intervention consultations as well as usage of the interventions by insured individuals, online-assessments, qualitative interviews and focus groups.

**Table 1:** RE-AIM dimensions, measures and assessments

Dimension	Measure	Source	Assessment type	Time point
**REACH (individual level)**	Number and characteristics of potentially eligible insured individuals	SVLFG	Routine data	18 m
	Exclusion, referral und uptake rates; Completer rating from clinician’s judgement (Helping Alliance Questionnaire)	GET.ON Institute / IVPNetworks / SVLFG; GET.ON and IVP coaches	Reportings; Online-Assessment	Every 3 months; after participant is defined as (non-) completer
	Estimated number of people exposed to the recruitment, recruitment response rate in general as well as specific recruiting strategies	GET.ON Institute / IVPNetworks / SVLFG	Reportings	Every 3 months
	Representativeness of participants compared to eligible insured individuals and interested, but not participating insured persons	SVLFG	Routine data	18 m
**EFFECTIVENESS (individual level)**	Depressive symptoms (Patient Health Questionnaire 9), perceived stress (Perceived Stress Scale 4), generalised anxiety disorder (Generalised Anxiety Disorder 7 scale), somatisation (Somatic Symptom Scale 8), health-related quality of life (Assessment of Quality of Life 4D), subjective capacity to work (Subjective Prognostic Employment Scale)	Insured person	Online-assessment	T0-T2^a^
	Patient satisfaction (Client Satisfaction Questionnaire adapted for internet interventions), side effects (Assessment of Negative Effects of Psychotherapy)	Insured person	Online-assessment	T1^a^
**ADOPTION (organisational level)**	Number, proportion and characteristics of involved employees per organisation, characteristics of federal states and rollout areas, characteristics of insured persons and type of agricultural holding	GET.ON Institute / IVPNetworks / SVLFG	Sociodemographics of employees, routine data	R0^b^ Continuous
**IMPLEMEN-TATION (individual + organisational level)**	*Characteristics of the interventions:*			
	- Adaptations (TIDieR checklist)	GET.ON Institute / IVPNetworks	Reportings	Mid and end of the project
	- Usage of the intervention (e.g. adherence, duration and frequency of contacts)	GET.ON Institute / IVPNetworks	Reportings	Continuous
	- Credibility (Credibility Expectancy Questionnaire) and perceived (dis-)advantages	SVLFG staff- participants	Online-assessment	R0 / R1 / R2 / R3^b^
	- Costs for the intervention	SVLFG	Reportings	Every 3 months
	*Outer Setting:*			
	Acceptance, relevance, utility	Insured person GET.ON Institute / IVPNetworks	Online-assessment Interviews Reportings (e.g. adherence)	T1^a^, T1^a^ Continuous
	*Inner Setting:*			
	- Leadership support (Implementation Leadership Scale)	SVLFG staff-participants	Online-assessment	R0 / R1^b^
	- Organisational willingness to change (Organisational Readiness for Implementing Change questionnaire)	SVLFG staff-participants, implementation team	Online-assessment	R0 / R1 / R2 / R3^b^
	*Characteristics of individuals:*			
	- Degree of normalisation (NoMAD)	SVLFG staff-participants	Online-assessment	R0 / R1 / R2 / R3^b^
	- Determinants of Behavioural Change (Discriminant Content Validation of the TDF Questionnaire)	SVLFG staff-participants)	Online-assessment	R1^b^
	- Barriers and facilitating factors at the consultations	SVLFG staff-participants	Focus groups	11/2017–02/2019
	*Process:*			
	- Evaluation of the implementation process (e.g. implementation activities, barriers and facilitating factors)	Implementation team	Interview	Every 6 months
	- Dissemination activities	SVLFG	Reportings	Every 3 months
	- Usage by employees	SVLFG staff-participants	Reportings	Every 3 months
	- Implementation costs	SVLFG	Reportings	Every 3 months
**MAINTENANCE (individual + organisatonal level)**	Sustainability of interventions (effectiveness)	Insured	Online-Assessment	T2^a^
	Referrals back to SVLFG and use of further offers	GET.ON Institute / IVPNetworks / SVLFG	Reportings	Continous
	Evaluation of the current implementation and further use of the technologies	SVLFG staff-participants	Online-assessment	R3^b^
	Sustainability of implementation (referral and uptake rates)	GET.ON Institute / IVPNetworks / SVLFG	Reportings	R3^b^

Notes

^a^time of insured-assessment depends on the date of intervention start

^b^time of employee-assessment depends on the date of the rollout in the specific area

*Abbreviation*: *m* month

Assessments: T0 (pre intervention), T1 (3 months after starting the internet-based training / 6 months after starting the tele-based intervention), T2 (12 months after starting the intervention)

Rollout: R0 (rollout start), R1 (6 months after rollout), R2 (12 months after rollout), R3 (18 months after rollout)

#### Reach

To assess reach, the number and characteristics of the potentially eligible insured individuals at SVLFG, as well as exclusion, referral and uptake rates of the internet-, tele-based and on-site group preventive services will be reported. The following uptake levels will be considered in order to illustrate different levels of engagement:

- Referral rates: The absolute number of: a) referrals by the SVLFG field workers (operationalised as consultations about internet- and tele-based interventions), b) referrals by the call centre agents to the internet- and tele-based prevention services (IMIs: sending out a code for the registration at the online-platform, tele-based coaching: registration on the IVPnet platform), and c) the first contact with the external service provider (IMIs: first log in at online-platform to symptom screening; tele-based coaching: agreement for first tele-based coaching appointment).
- Uptake rates of internet- and tele-based interventions:
  - Intervention not started: The absolute number of referred persons who completed the assessment phase (IMIs: psycho-diagnostic assessment, tele-based coaching: first coaching assessment session) after the first contact with the external service provider, but have not started the intervention.
  - In treatment: The absolute number of persons who have started the intervention after assessment phase (IMIs: first login into first module; tele-based coaching: receive tele-based coaching at minimum second session).
  - Treatment not completed: Absolute number of persons who completed less than 50% vs. 70% of the intervention (IMIs: completed less than 50% vs. 70% maximal online modules within 13 weeks, have not logged in for at least 8 weeks or have interrupted the training for other reasons; tele-based coaching: completed less than 50% vs. 70% of the total call minutes, did not answer for at least 8 weeks or cancelled the coaching for other reasons).
  - Treatment completed: a) In previous e-mental health studies, the completer criteria differed between 50 to 100% of completed units [81–84]. Research also indicates that a minimal treatment for subthreshold depression (e.g. six phone calls each with a maximum of 15 min) can also be effective in preventing MD [85]. Therefore, we defined two criteria with different treatment dosis as following: Absolute number of persons who completed at least 50% vs. 70% of online modules or call minutes. b) Clinical judgment by coaches at GET.ON Institute and IVPNetworks assessed with the Helping Alliance Questionnaire (HAQ) based on 11 items [86] in order to supplement the quantitative criteria for completion.
- Uptake of other services: This may include the crisis hotline, a second guided IMI, diagnostic clarification at IVPNetworks (via telephone or on-site), referrals to social or health service centres (e.g. socioeconomic or family consultants) during the tele-based coaching.

In addition, the estimated number of people exposed to recruitment materials and the associated response rate will be assessed. Specific recruiting strategies are tested to examine successful ways of addressing the target population. To provide representative information, interested but not participating individuals, and enrolled participants will be compared based on routine data. Differences in reach between the internet-, tele-based and on-site preventive services will be investigated as well.

#### Effectiveness

Effectiveness will be assessed at pre- and post-treatment (3 months after first log in on the GET.ON platform or 6 months after the registration at IVPnet) with online surveys consisting of frequently used and well validated self-report questionnaires. Depressive symptoms are assessed using the German Version of the Patient Health Questionnaire 9 (PHQ-9) [87] with high values on internal consistency (α = 0.88) [88]. Generalised Anxiety Disorder severity is measured by the Generalised Anxiety Disorder 7 item self-report scale (GAD-7; α = 0.89) [89, 90]. Perceived stress is assessed with the German Version of Perceived Stress Scale with 4 items (PSS-4; α = 0.84) [91]. Somatisation tendencies will be measured by using the Somatic Symptom Scale based on 8 items (SSS-8; α = 0.81) [92, 93]. The 12-item Assessment of Quality of Life instrument (AQoL–4D; α = 0.81) [94] will be used to assess health-related quality of life. Subjective capacity to work will be assessed by using the Subjective Prognostic Employment Scale (SPE; Guttman scaling: rep = 0.99) based on 3 items [95]. Side effects attributed to the intervention are collected in the post-assessment period by the Inventory for the Assessment of Negative Effects of Psychotherapy (INEP; 22 items; α = 0.86) [96] adapted to internet- and tele-based interventions.

#### Adoption

Adoption will be assessed through the number and characteristics of the involved employees at SVLFG as well as at the external provider (GET.ON institute, IVPNetworks). Furthermore, metrics of the rollout regions and federal states, such as amount and characteristics of insured people and type of agricultural holdings will be described and compared to one another.

#### Implementation

After a broad literature search, questionnaires were selected that fit the study context as best as possible (e.g. can also be used for employees who are involved in the referral and not in the service delivery itself).

##### Intervention characteristics

Potential adaptations to the existing interventions during the implementation process will be reported by the external service provider following the TIDieR checklist [51] halfway through the implementation and when concluding the project. Adaptations in the course of the implementation relate to referral and registration ways (e.g. to overcome barriers to participation). Furthermore, stakeholders’ (e.g. field workers, in-house staff, call centre agents) perception of perceived (dis-)advantages of internet- and tele-based interventions and the treatment credibility will be assessed using the Credibility Expectancy Questionnaire (CEQ) with 6 items [96]. The psychometric properties of the CEQ are characterized by high internal consistency (α = 0.84–0.85) and good values on test-retest reliability [97]. Intervention costs are recorded during the study as cost per participant for the respective intervention. Likewise, the use of the interventions by the insured individuals (including adherence, duration of intervention, number and duration of contacts, main topics, therapeutic background of coaches, etc.) will be monitored.

##### Outer setting

Constructs including participants’ needs and resources are related to the CFIR dimension ‘outer setting’ [43]. In assessing the outer setting, participant satisfaction is measured by using a German Version of the Client Satisfaction Questionnaire adapted for internet interventions (CSQ-I; α = 0.93) based on 8 items [65, 98]. The participants are asked about the usefulness and relevance of the interventions. Acceptance of the interventions is indirectly recorded via non-completer rates (indicator, see above). Approximately 20 semi-structured interviews each with participants from GET.ON chronic pain, from the six remaining IMIs and from the tele-based coaching will supplement the data collection and provide insights into the participants’ experience (e.g. acceptance, satisfaction, barriers and facilitators for intervention usage) with the internet- or tele-based interventions. The interview guide is based on the Unified Theory of Acceptance and Use of Technology (UTAUT) model [99] and the Discrepancy Theory of Satisfaction [100] with regards to the dimensions: technical quality of care, psychosocial quality of care, organisational conditions, spatial and technical equipment, treatment outcome, continuity of care and financing [100].

##### Inner setting

Constructs such as organisational culture and leadership engagement are related to the CFIR dimension ‘inner setting’ [43]. In assessing the inner setting, organisational readiness for implementing change for field workers, in-house staff, call centre agents and the implementation team at SVLFG is measured by using a German version of the Organisational Readiness for Implementing Change (ORIC) questionnaire with 12 items [101]. The ORIC questionnaire focuses on the degree to which employees are psychologically and behaviorally ready to implement organizational changes, which can be considered as a decisive factor for implementation success [101]. Good internal consistency (α = 0.88–0.92) is reported for the ORIC questionnaire [101]. Leadership of the implementation team as well as of the supervisors of field workers and call centre agents at SVLFG will be captured by using the Implementation Leadership Scale (ILS) based on 12 items [102]. The ILS can be seen as an efficient and short questionnaire which has been validated in settings of mental health with very high values on internal consistency (α = 0.93–0.97) [103].

##### Characteristics of the individuals

The CFIR dimension of ‘characteristics of the individuals’ encompasses knowledge and beliefs about the intervention, self-efficacy and other personal attributes of the staff participants at SVLFG [43]. The degree of normalisation among staff-participants (e.g. field workers, in-house staff, call centre agents) is assessed by the German translation of the NoMAD and 3 additional normalization questions; the questionnaire (20 items) refers to the normalisation process theory and assesses the extent to which the new implemented service is a normal part of the daily routine at work [104]. The NoMAD questionnaire is characterized by a high internal consistency (α = 0.89) and has already been validated in heterogeneous samples [105, 106]. Determinants of behavioural change (e.g. knowledge, belief, self-efficacy) are assessed with the Discriminant Content Validation of the TDF Questionnaire (DCV) with 32 items [107] among field workers, in-house staff and call centre agents. The DCV helps to understand factors that influence the behaviour in health care settings and can therefore makes it possible to improve the implementation of innovations. Additionally, about 20 focus groups using an interview guide based on TDF [45] with field workers are conducted to identify barriers and facilitating factors for advising on prevention services. The aim of this mixed-method approach is the optimisation of the implementation process or interventions based on the findings.

##### Implementation process

To capture the implementation process, the number and type of implementation and dissemination activities of SVLFG, GET.ON and IVP are reported every 3 months with an excel file (e.g. date, place, organisation, setting of the activity, target group, number of participants and content / aim of the activity). Implementation costs are reported every 3 months with an excel file and are defined according to the EU-project ImpleMentAll^2^ project as the sum of personnel costs and other direct and indirect costs (indirect costs calculated at 20% of direct costs). In addition, the implementation team is interviewed every 6 months, particularly with regards to perceived effectiveness of the implementation in general and based on individual implementation strategies. These interviews also discuss barriers and facilitating factors in the implementation process and assessment of the current state of implementation. Quantitative (referral and uptake rates, barriers and facilitating factors in the implementation) and qualitative results (focus groups) about the progress and quality of implementation are prepared and delivered to the implementation team every 6 months to promote adaptions and improvements.

#### Maintenance

In assessing the maintenance of the implemented internet- and tele-based interventions, follow-up surveys with the insured participants will be conducted 12 months after first contact for the prevention project (e.g. 12 months after first login at the GET.ON platform or registration at IVPnet) to monitor the long-term impact of preventive interventions on mental health (see effectiveness measures, 60–65). Referrals to the SVLFG call centre are reported by the external service providers to capture all processes in the implementation that contribute to sustainable development. Uptake rates of the internet- and tele-based interventions are monitored in the maintenance phase of the respective rollout region. In terms of sustainability, we also record the use of a second IMI, the maintenance phase of the IMIs (e.g. monthly contacts for a year) or the prolongation of an IVP tele-based coaching and any other health care utilisation.

Normalisation is recorded in the maintenance phase using the questionnaire NoMAD [104] at the staff-level (e.g. field workers, in-house staff, call centre agents), and will be compared to the normalisation degree during the implementation phase. Additionally, the attitudes and experiences of the staff-participants involved in the implementation process are essential for sustained implementation. Therefore, a concluding survey with the staff-participants and important multipliers at SVLFG (e.g. management board) will be conducted at the end of the project to assess their professional view on the implementation and their intention to further use the internet- and tele-based prevention services. To ensure practical relevance, the item pool for this survey will go through an iterative feedback process with the initiators of the project.

#### Measuring implementation success

In line with the ImpleMentAll,^3^ implementation success is defined as an increase in uptake of internet- and tele-based interventions (referral rates and levels of uptake, e.g. not started, in treatment, completer, non-completer), degree of normalisation among staff-participants (extent to which consultation to the internet- and tele-based interventions are perceived as a normal part of work) and efficiency outcomes (costs of implementation effort in relation to uptake and degree of normalisation).

### Analysis

Reporting of implementation results will follow the StaRI [49] statement. To assess the implementation success and to illustrate a broad picture of the implementation process in the context of rural areas we will combine qualitative and quantitative methods in a summative way. Quantitative and qualitative data are collected simultaneously to evaluate the implementation process. Qualitative data are used to provide a deep understanding, while quantitative data are used to provide breadth of understanding [108]. In addition, the results of the qualitative analysis are then validated in a quantitative survey. Qualitative surveys are only carried out on a small part of the sample, while all participants are invited to the respective quantitative surveys. The database is therefore larger for quantitative surveys. The analysis is based on guidelines for mixed-methods research defined by the NIH Office of Behavioural and Social Sciences Research [109]. In addition, the Consolidated criteria for reporting qualitative studies (COREQ) [110] are used.

#### Quantitative analysis

The evaluation of the implementation follows the RE-AIM dimensions as described above. Descriptive analyses (frequencies, means and percentages) will be performed to assess outcomes across all RE-AIM dimensions. We will evaluate the implementation success of the internet- and tele-based interventions separately.

To assess the representativeness of the participants compared to the target group, t-tests or non-parametric tests will be used. To assess intervention effectiveness, we analyse for different groups of participants (e.g. participants who have started treatment or completed treatment) by using independent sample t-test and chi-squared analyses. We will use t-tests or non-parametric tests to analyse differences between pre-, post- and follow-up-measurements. In order to counteract the issue of multiple comparisons, Bonferroni correction is used. The statistical significance level is set to *p* < .05. Within-group effect sizes and 95% CIs will be reported at all measurement points. Implementation effectiveness is assessed by comparing uptake rates, the degree of normalisation among staff-participants and efficiency outcomes (costs of implementation effort in relation to uptake and degree of normalisation) at different time points.^4^

Furthermore, linear regression models are used to investigate differences between the interventions (guided IMIs, personalised tele-based coaching and on-site group workshops) regarding reach outcomes (referral and uptake rates) with intervention type as independent variable. By using further linear regression models we compare socio-demographic and clinical outcomes as dependent variables between the participants of the different prevention services (independent variable). Based on multiple linear regression models we analyse the influence of factors at staff level (credibility of the internet- and tele-based interventions, degree of normalisation, leadership support, organisational readiness to change and determinants of behavioural change) as independent variables on the number of referrals by field workers, in-house staff and call centre agents (dependent variables). Additionally, the implementation outcomes will be compared between the federal states and rollout areas at different phases of implementation.

According the intention-to-treat principle all observed data will be included. Patterns of missing data will be analysed and corrected. All analyses will be performed with R statistic software [111].

#### Qualitative analysis

Qualitative data is used for insights into the implementation process. Focus groups with field workers are conducted to understand barriers and facilitating factors of the consultation regarding the internet- and tele-based services. Interviews with insured persons enable insights in participants’ experience (e.g. satisfaction, acceptance). All qualitative data will be audiotaped and transcribed verbatim using MAXQDA. The analysis is based on the qualitative content analysis by Mayring [112]. To establish reliability, two independent raters will code the same transcripts using a coding guide and coding rules. The focus groups are evaluated inductively as well as deductively, based on the TDF [45] in two different coding passes. The interviews with the insured individuals are evaluated with an inductive-deductive approach.

## Discussion

This study provides novel and diverse information to the existing literature regarding the implementation of preventive treatments for depression. To our knowledge, this study is the first nationwide prevention project in Germany and the first comprehensive and multi-level implementation study in general that examines the implementation of internet- and tele-based interventions in farmers, gardeners and foresters in rural areas. The implementation process of different preventive services such as internet- and tele-based interventions will be compared. Insights into implementation of mental health services on an individual (participants, staff) and organisational level, within the context of rural areas, can be assessed. Additionally, the associated costs (implementation and intervention costs) will be considered. Furthermore, determinants for implementation success in rural areas can be identified by focusing on employees involved in the referral process. Based on these findings, recommendations for future interventions can be made.

The three parallel running RCTs [40, 41]^5^ will deliver results on the clinical effectiveness of the internet and tele-based interventions under randomized controlled conditions. Results of the implementation study will provide information about the implementation of the internet- and tele-based interventions from a routine care perspective in terms of a hybrid study design simultaneously examining the impact of the interventions in routine care (e.g. pre-, post- and follow-up comparisons at participant level) and the implementation itself. This enables results based on two different, complementary study designs. The results will show to what extent the implementation of technologies as preventive offers will be accepted by the participants as well as employees involved in the implementation process. If the implementation of the internet- and tele-based services succeeds, these services may be feasible in the long-term and health care in rural areas could be sustainably improved.

### Trial status

Recruitment has been started in April 2019. Participants will be continuously included until the end of the study. Data collection is ongoing.

## Supplementary information

**Additional file 1.** Standards for Reporting Implementation Studies: the StaRI checklist for completion

**Additional file 2.** Description of the GET.ON online health trainings

**Additional file 3.** Description of the personalised tele-based coaching (IVPNetworks)

## Data Availability

Results will be submitted for publication in a peer-reviewed journal and presented at conferences. Central results will be communicated to SVLFG and can be used to better understand insured persons, disseminate the information in health care campaigns and further improve health care services for farmers, foresters and gardeners. Access to the final trial dataset can be provided to fellow researchers upon request, depending on to be specified data security and data exchange regulation agreements.

## Footnotes

1 Terhorst, Y., Braun, L., Titzler, I., Buntrock, C., Freund, J., Thielecke, J., Ebert, D. D., Baumeister H. Clinical and cost-effectiveness of a guided internet-based Acceptance and Commitment Therapy to improve chronic pain related disability in green professions (PACT-A): study protocol of a 36-month follow up pragmatic randomized controlled trial (under review).

2 Bührmann L, Schuurmans J, Ruwaard J, Genugten C van, Finch T, Rapley T, et al. Tailoring Implementation of Internet-based Cognitive Behavioural Therapy to Context-specific Needs across Europe and Australia. A Study Protocol for a Stepped-wedge Cluster Randomized Trial: the ImpleMentAll Project (under review).

3 Bührmann L, Schuurmans J, Ruwaard J, Genugten C van, Finch T, Rapley T, et al. Tailoring Implementation of Internet-based Cognitive Behavioural Therapy to Context-specific Needs across Europe and Australia. A Study Protocol for a Stepped-wedge Cluster Randomized Trial: the ImpleMentAll Project (under review).

4 Bührmann L, Schuurmans J, Ruwaard J, Genugten C van, Finch T, Rapley T, et al. Tailoring Implementation of Internet-based Cognitive Behavioural Therapy to Context-specific Needs across Europe and Australia. A Study Protocol for a Stepped-wedge Cluster Randomized Trial: the ImpleMentAll Project (under review).

5 Terhorst, Y., Braun, L., Titzler, I., Buntrock, C., Freund, J., Thielecke, J., Ebert, D. D., Baumeister H. Clinical and cost-effectiveness of a guided internet-based Acceptance and Commitment Therapy to improve chronic pain related disability in green professions (PACT-A): study protocol of a 36-month follow up pragmatic randomized controlled trial (under review).
