# Supplementary material for: Implementing internet- and tele-based interventions to prevent mental health disorders in farmers, foresters and gardeners (ImplementIT): study protocol for the multi-level evaluation of a nationwide project
Source: BMC Psychiatry. 2020 Aug 27;20:424. doi: 10.1186/s12888-020-02800-z (PMC7450981; doi:10.1186/s12888-020-02800-z)
Supplement: Supplementary file 2 — Additional file 2. Description of the GET.ON online health trainings [file 12888_2020_2800_MOESM2_ESM.docx]

## Additional file 2: Description of the GET.ON online health trainings

1. **BRIEF NAME:** *Provide the name or a phrase that describes the intervention.*

GET.ON online health trainings

1. **WHY:** *Describe any rationale, theory, or goal of the elements essential to the intervention.*

The GET.ON online health trainings are primarily based on cognitive behavioural therapy (CBT) and contain elements of systemic therapy, and acceptance and commitment therapy, except for the training GET.ON Stress, which was based on transactional model of stress and coping.The goal of the trainings is to decrease mental health issues, in particular depressive symptoms.

1. **WHAT: Materials:** *Describe any physical or informational materials used in the intervention, including those provided to participants or used in intervention delivery or in training of intervention providers. Provide information on where the materials can be accessed (e.g. online appendix, URL).*

The intervention consists of seven trainings: *GET.ON Mood Enhancer*, *GET.ON Stress*, *GET.ON Recovery*, *GET.ON Mood Enhancer Diabetes*, *GET.ON Panic*, *GET.ON Chronic Pain* and *GET.ON Be smart – drink less*. All trainings use materials such as audio and video clips, exercises, diaries and mock individuals. Trainings approach specific problems relevant for farmers, foresters and gardeners.

The coaches are provided with general guidelines and training manuals.

1. **WHAT: Procedures:** *Describe each of the procedures, activities, and/or processes used in the intervention, including any enabling or support activities.*

The insured person receives a GET.ON code from the SVLFG call centre, which enables him/her to register on the online platform. To start the intervention phase, the participant completes a computer-adaptive psycho-diagnostic assessment which is based on self-report questionnaires assessing depressive symptoms using the Patient Health Questionnaire (PHQ-8), perceived stress with the Perceived Stress Scale (PSS-10) and severity of insomnia with the Insomnia Severity Index (ISI). Additional constructs are measured including chronic pain assessed by the German version of the Impairment Scale of the Multidimensional Pain Inventory (MPI-D), harmful alcohol drinking by using the Alcohol Use Disorders Identification Test-Consumption (AUDIT-C) as well as panic and agoraphobic symptoms with the Panic and Agoraphobia Scale (PAS). The type of online training is chosen during the first interview with the e-coach depending on the individual’s symptom profile and the needs and preferences of the participant.

Then the participant receives to the chosen training. It is recommended that participants complete one module per week. During this active training phase, the coach is available to answer questions as they arise and provides feedback to the participants after each module. The participant can choose between feedback via e-mail or telephone. After receiving the feedback, the participants can start the next module. During the maintenance phase, there are monthly contacts between the participant and the coach.

Coaches are responsible for the first contact, reminding participants to complete modules, evaluating the assessment, facilitating the active training and maintaining contact with participants during the maintenance phase. GET.ON support is also available to participants, if technical problems arise.

1. **WHO PROVIDED:** *For each category of intervention provider (e.g. psychologist, nursing assistant), describe their expertise, background and any specific training given.*

The coaches are psychologists with a university degree who have at least started a psychotherapeutic training. They receive supervision by licensed psychotherapists in CBT training.

The training of the coaches consists of:

1. Self-experience
2. Regular trainings
3. Supervision and intervision
4. Information about IT-matters
5. **HOW:** *Describe the modes of delivery (e.g. face-to-face or by some other mechanism, such as internet or telephone) of the intervention and whether it was provided individually or in a group.*

The training is provided individually. The lectures during the active phase are internet-based. The participant can choose between written (via e-mail) or oral (via telephone) feedback by the GET.ON coach. In the following maintenance phase, there are monthly coaching contacts via e-mail or telephone.

1. **WHERE:** *Describe the type(s) of location(s) where the intervention occurred, including any necessary infrastructure or relevant features.*

There is no requirement for specific locations. The coaching can be administered on any device that is able to connect to the internet. Furthermore, an e-mail address is necessary.

1. **WHEN AND HOW MUCH:** *Describe the number of times the intervention was delivered and over what period of time including the number of sessions, their schedule, and their duration, intensity or dose.*

Each of the seven online training consists of 6-8 module (GET.ON Stress: 7 standard modules; GET.ON Be smart - drink less: 8 standard modules; Other: 6 standard modules) with psychoeducation and in-depth exercises to improve mental health symptoms with different contents (i.e. content regarding depressed mood in general or comorbid with diabetes mellitus, insomnia, stress, panic and agoraphobic symptoms, chronic pain and harmful alcohol use). After completion of the training with an estimated duration of 6-8 weeks, a maintenance phase takes place consisting of up to 12 follow up months. If needed, additional training can be administered.

1. **TAILORING:** *If the intervention was planned to be personalised, titrated or adapted, then describe what, why, when, and how.*

The online trainings are individually tailored to the participants with regard to their symptoms, risk profile and needs. The different trainings address risk factors for the incidence of depression including subclinical depressive symptoms, insomnia, stress, anxiety, chronic pain, harmful alcohol use and subclinical depressive symptoms in the context of diabetes. Before starting a training, the participant completes a computer-adaptive psycho-diagnostic assessment to determine which training fits best for them.

During the active training phase the participant can choose between feedback via e-mail or via telephone after each module.

In order to establish relevant problems, interviews with farmers, foresters and gardeners were completed and different types of agricultural holdings were visited. The content and design of the trainings was then adapted to the target group of farmers, foresters and gardeners to meet the needs of the target group.

1. **MODIFICATIONS:** *If the intervention was modified during the course of the study, describe the changes (what, why, when, and how).*

Not applicable.

1. **HOW WELL:** *Planned: If intervention adherence or fidelity was assessed, describe how and by whom, and if any strategies were used to maintain or improve fidelity, describe them.*

The quality management system of the GET.ON institute includes following aspects:

- Standards for trainings
- Standards for qualifications and continuous support of the coaches
- Standards for knowledge transfer
- Standards for continuous optimisation of the processes
- Standards for the guarantee of following legal and contract settlements
- Standards for a report system

Additionally, the Friedrich-Alexander University Erlangen-Nürnberg monitors and analyses adherence and usage of the intervention.

1. **HOW WELL:** *Actual: If intervention adherence or fidelity was assessed, describe the extent to which the intervention was delivered as planned.*

Not applicable.
