# Supplementary material for: Implementing internet- and tele-based interventions to prevent mental health disorders in farmers, foresters and gardeners (ImplementIT): study protocol for the multi-level evaluation of a nationwide project
Source: BMC Psychiatry. 2020 Aug 27;20:424. doi: 10.1186/s12888-020-02800-z (PMC7450981; doi:10.1186/s12888-020-02800-z)
Supplement: Supplementary file 3 — Additional file 3. Description of the personalised tele-based coaching (IVPNetworks) [file 12888_2020_2800_MOESM3_ESM.docx]

## Additional file 3: Description of the personalised tele-based coaching (IVPNetworks)

1. **BRIEF NAME:** *Provide the name or a phrase that describes the intervention.*

Personalised tele-based coaching (IVPNetworks)

1. **WHY:** *Describe any rationale, theory, or goal of the elements essential to the intervention.*

The personalised tele-based coaching focuses on general mental health problems. The primary goal of tele-based coaching is to reduce mental illness. Therefore, the coaches support participants in recognising and understanding conflict patterns so that they can effectively manage them by activating their own resources and coping strategies. This is pursued by approaching the participant’s personal situation and stressors (e.g., financial burden, family problems, work-related stress). Personalised coaching means that there are no standardised manuals or fixed procedures for the coaching. Depending on the therapeutic background of the coach, elements of hypnotherapy, systemic, cognitive behavioural or psychodynamic therapy, etc. may be used.

1. **WHAT: Materials:** *Describe any physical or informational materials used in the intervention, including those provided to participants or used in intervention delivery or in training of intervention providers. Provide information on where the materials can be accessed (e.g. online appendix, URL).*

- The participant receives a business card with the coach’s contact information for emergencies.
- Fitting their individual situation, participants can receive materials with diverse and specific psychoeducation information, which may contain coping strategies or specific exercises.

1. **WHAT: Procedures:** *Describe each of the procedures, activities, and/or processes used in the intervention, including any enabling or support activities.*

The call centre of the SVLFG registers the insured individuals on the online-platform of IVPNetworks (IVPnet 2.0). The coaches are matched with participants based on the participant’s needs as well as the coach’s experience. The participant is contacted via e-mail or mail by the IVP case management and receives the contact information (i.e. business card) for their coach. Afterwards, the coach will contact the participant to arrange a first appointment.

During the coaching, coaches interactively work with participants towards solution-oriented improvements of their current situation. Every talk begins with a “warm up“ and a conversation about achieving recent goals or tasks before new topics are addressed. If indicated, participants are supported in finding on-site social and health care services to complement the tele-based coaching (e.g., socioeconomic consultants, agricultural family counselling).

Alternatively, on-site coaching can be arranged if a participant no longer prefers tele-based coaching. In the case that a participant’s depressive symptoms worsen at any time during the coaching, further steps will be arranged to support the referral into routine care.

1. **WHO PROVIDED:** *For each category of intervention provider (e.g. psychologist, nursing assistant), describe their expertise, background and any specific training given.*

The coaches at IVPNetworks are psychologists with university degree in psychology who have completed a training in cognitive-behavioural therapy, systemic therapy, psychodynamic therapy, hypnotherapy, etc. All 20-30 coaches are managed by IVPNetworks. The coaches are either employed by IVPNetworks or self-employed. Licensed psychotherapists are available for supervision.

**HOW:** *Describe the modes of delivery (e.g. face-to-face or by some other mechanism, such as internet or telephone) of the intervention and whether it was provided individually or in a group.*

The coaching is provided individually and carried out via telephone.

1. **WHERE:** *Describe the type(s) of location(s) where the intervention occurred, including any necessary infrastructure or relevant features.*

There is no requirement for specific locations for training, the coaching can be carried out in any location with phone reception.

1. **WHEN AND HOW MUCH:** *Describe the number of times the intervention was delivered and over what period of time including the number of sessions, their schedule, and their duration, intensity or dose.*

The duration, frequency and length of the coaching is individualised with a maximum of 850 minutes, distributed in flexible sessions of either 25 or 50 minutes over three to six months. If needed, the coaching can be prolonged up to nine months (resulting in up to additional 150 minutes).

1. **TAILORING:** *If the intervention was planned to be personalised, titrated or adapted, then describe what, why, when, and how.*

Prior to interacting with any study participants, the coaches receive an introduction into common problems faced by farmers, foresters and gardeners. Informational materials on agricultural topics and on-site preventive services are provided by SVLFG.

At the beginning of the coaching, individual goals for the participants are established. The coaching is personalised in length, duration and content.

1. **MODIFICATIONS:** *If the intervention was modified during the course of the study, describe the changes (what, why, when, and how).*

Not applicable.

1. **HOW WELL:** *Planned: If intervention adherence or fidelity was assessed, describe how and by whom, and if any strategies were used to maintain or improve fidelity, describe them.*

Durance, length and content of the conversations, coaching topics, referral to on-site social and health care services, dropout, etc. are documented by the coach in the patient files of IVPnet. The Friedrich-Alexander University Erlangen-Nürnberg is responsible for the evaluation of this information.

1. **HOW WELL:** *Actual: If intervention adherence or fidelity was assessed, describe the extent to which the intervention was delivered as planned.*

Not applicable.
